# Finite Element Analysis of Electromagnetic Characteristics of a Single-Phase Permanent Magnet Linear Oscillation Actuator

**DOI:** 10.3390/s25020452

**Published:** 2025-01-14

**Authors:** Hongbin Zhang, Zhaoxin Wang, Minshuo Chen, Zhan Shen, Haitao Yu, Zhike Xu

**Affiliations:** 1 Jiangsu Maritime Institute, Nanjing 210096, China; 230208301@seu.edu.cn; 2 AAU Energy, Aalborg University, Aalborg 9220, Denmark; 3 Advanced Ocean Institute of Southeast University, Nantong 226010, China; chenminshuo@seu.edu.cn; 4 Department of Electrical Engineering, Southeast University, Nanjing 210096, China; zhs@seu.edu.cn (Z.S.); htyu@seu.edu.cn (H.Y.); xuzhike@seu.edu.cn (Z.X.)

**Keywords:** linear oscillation actuator, finite element methods, permanent magnet, electromagnetic performance

## Abstract

The electromagnetic characteristics of a single-phase permanent magnet linear oscillation actuator are analyzed by the finite element method. Firstly, the basic structure and operation principle of the linear oscillation actuator are introduced. The internal stator slot and arc tooth are used to reduce the detent force. According to the principle of electromagnetic fields, the electromagnetic field equation is listed and the function of the motor is deduced. At the same time, the eight-node hexahedral element is used to calculate the listed universal functions, and the inductance, flux linkage, induced electromotive force and electromagnetic force of the motor are deduced. The electromagnetic field of the motor is simulated by two-dimensional and three-dimensional finite element methods, and the accuracy of the calculation results of the electromagnetic characteristics of the cylindrical linear oscillation motor by the two methods is compared and analyzed. Finally, an experimental prototype was developed and the no-load characteristics of the motor were tested using the existing linear motor towing method. By comparing the experimental and simulation results, the accuracy of the theoretical analysis and the rationality of the motor design are verified.

## 1. Introduction

A linear compressor, driven directly by a linear oscillating actuator (LOA), can greatly improve the efficiency of a refrigeration system, while reducing the overall volume. Compared with traditional three-phase LOAs, single-phase permanent magnet linear oscillation actuators (SPMLOAs) have the advantages of simple mechanical structure, low manufacturing cost, fast reaction speed, and no motion conversion device, and are widely used in short stroke high-frequency places [1,2]. Usually, the SPMLOAs adopt cylindrical structures, which can effectively reduce end windings and achieve higher air gap magnetic density and efficiency. Compared with the E-type stator core, the C-type stator core has a simpler structure and relatively easier assembly process, but at the same time, the output thrust coefficient is smaller [3]. Therefore, in order to increase the output thrust coefficient of this structure, many researchers have adopted the method of increasing the number of permanent magnets. However, these measures also generate significant detent force, thereby affecting the smooth operation of the LOA system.

The combination of ferrite and permanent magnets in LOA can improve the output thrust coefficient without increasing the cost of the LOA [4]. However, the installation position of ferrite can lead to an increase in the axial length of the LOA, which limits its application in environments with strict space constraints. In order to eliminate the detent force of the linear motors, various optimization techniques have been applied to traditional permanent magnet linear motors (PMLMs) [5,6,7,8,9]. Due to the fact that the detent force in PMLMs is generated by the interaction between permanent magnets and the stator teeth, it is mainly divided into cogging force and end force. Therefore, in reference [5], oblique slot and stepped end tooth methods are used to eliminate cogging force and end force, respectively. At the same time, improving the smoothness of the air gap magnetic flux density can also reduce the detent force. Therefore, for the teeth of PMLMs, arc-shaped [6], chamfered [7], and stepped [8] designs can be used to suppress the detent force. However, there is limited research on suppressing the detent force of SPMLOAs, and there are few articles evaluating it. Due to the unique structure and motion modes of SPMLOAs, the detent force that exhibits linear changes within the effective formation can be equivalent to a magnetic spring, and therefore its impact on electromagnetic performance can be ignored [9]. Although this method has certain advantages, it limits the variation of the effective stroke of the LOA and increases the difficulty of spring selection [10].

The most widely used methods for calculating the magnetic field of the motor are analytical and numerical methods. The electromagnetic field analysis method is based on the basic theory of electromagnetic fields, establishing partial differential equations for each part of the motor, and solving them by combining boundary conditions and the constitutive relationship of the medium. When the motor structure is complex or saturation problems need to be considered, further precise subdomains need to be constructed for calculation. Therefore, the analytical method is more complex, and the number of equations and variables increases with the number of subdomains. Electromagnetic field numerical calculation is based on the electromagnetic field analysis method, which discretizes continuous mathematical models and combines them with computer programs for numerical solution. The finite element method (FEM) has strong adaptability in numerical calculation methods and can effectively handle complex electromagnetic structures and nonlinear problems, making it the most widely used [11,12,13]. Reference [11] analyzed the characteristics of a U-shaped permanent magnet switch flux linear motor using FEM. Through comparative analysis, it has been proven that linear motors with U-shaped stators have higher thrust density and structural strength. Reference [12] combined this with a practical application environment, analyzed the secondary segmented linear flux switching permanent magnet motor using two-dimensional finite element analysis (2-D FEA), and verified the rationality of the motor design. Reference [13] accurately modeled a cylindrical permanent magnet synchronous linear motor and verified the correctness of the model through FEA and prototype testing. Meanwhile, for the study of cylindrical transverse flux switching linear motors, reference [14] established a mathematical model of the motor using an equivalent magnetic circuit, and calculated the motor magnetic field through 2-D and three-dimensional (3-D) finite element simulations, comparing it with traditional cylindrical primary permanent magnet linear motors. Reference [15] proposed a novel stator permanent magnet type moving iron core transverse flux linear oscillation motor, and optimized the dimensional parameters using analytical methods. The accuracy of the optimization results was verified using 3-D FEM.

In the field of high-power and high-frequency linear compressors, although existing linear oscillation motors can be applied, they still have above-mentioned problems [16]. Therefore, this article proposes a new topology structure for traditional cylindrical single-phase permanent magnet linear oscillation motor, which is innovative in that auxiliary slots are set at specific positions on the inner stator, and the stator teeth are treated with arc shape to effectively reduce positioning force. This article first introduces the basic structure of a linear oscillation motor, which uses internal stator slots and arc-shaped teeth to reduce positioning force. According to the principle of electromagnetic field, the electromagnetic field equation is listed and the functional of the motor is derived. At the same time, this article used an eight-node hexahedral element to calculate the listed universal functions and derived the inductance, magnetic flux, induced electromotive force (EMF), and electromagnetic force of the motor. The electromagnetic field of the motor was simulated and calculated using 2-D and 3-D FEMs, and the accuracy of the calculation results of the electromagnetic characteristics of the cylindrical linear oscillation motor was compared and analyzed between the two methods. Finally, an experimental prototype was developed to test the no-load characteristics of a linear oscillating motor using the existing linear motor towing method. By comparing experimental and simulation results, the accuracy of theoretical analysis and the rationality of motor design have been verified.

## 2. Motor Structure Analysis

**Figure 1:**
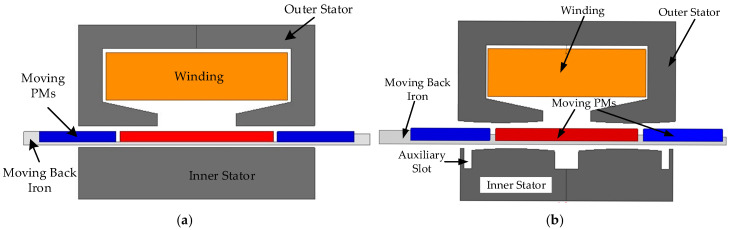
Structures of the two SPMLOAs: (**a**) traditional LOA; (**b**) novel LOA.

The topology structures of the two SPMLOAs are shown in Figure 1. The traditional SPMLOA shown in Figure 1a adopts a C-shaped iron core structure for the outer stator. This semi-open slot structure can maximize the effective slot area, but it also increases the difficulty of the winding assembly. Therefore, when assembling the outer stator, the tooth and yoke parts are usually separated. The mover of the SPMLOA is composed of a combination of permanent magnets and non-magnetic back iron. Compared to a single permanent magnet, the mover with three permanent magnet structures can provide a larger magnetic load in the main magnetic circuit, while reducing the magnetic resistance in the magnetic circuit, effectively increasing the output thrust coefficient of permanent magnets of the same mass. The stator inside the LOA is composed only of silicon steel sheets, mainly providing a magnetic circuit function for the magnetic flux of the mover permanent magnet. This structure can effectively reduce the thickness of the back iron of the mover, thereby reducing the mass of the mover and increasing the resonance frequency of the LOA. Figure 1b shows the structure of the SPMLOA with inner stator arc teeth and the auxiliary slot structure proposed in this paper. Compared to a traditional LOA, this structure is equipped with auxiliary slots in the stator, which can cancel each other out using end forces and cogging forces. By introducing new cogging forces, the detent force of the SPMLOA can be reduced. The arc-shaped tooth structure can smooth the changes in air gap magnetic resistance to reduce abrupt changes, and play a role in suppressing detent force.

## 3. Electromagnetic Field Equation and Finite Element Calculation

At present, the use of 2-D FEM to calculate the performance of the SPMLOA has been widely applied. However, due to the special structure of the cylindrical SPMLOA studied in this paper, the accuracy of the calculation results cannot be guaranteed by using 2-D calculation. Therefore, this paper adopts 3-D FEM to calculate the model.

### 3.1. Electromagnetic Field Equation

For the calculation of the SPMLOA magnetic field, FEA of the 3-D eddy current field is required to calculate the steady-state performance of the motor, based on Maxwell equations and the independent vector equations obtained in [17]:

(1) $$\left\{\begin{array}{c} \nabla \times H=J+\frac{\partial D}{\partial t}\\ \nabla \cdot B=0 \end{array}\right.$$

(2) B=μH

(3) J=σE

where H represents magnetic field strength, J is current density, D is potential shift, B is magnetic induction strength, μ is magnetic permeability, and σ is electrical conductivity. Due to the low frequency of the time-varying magnetic field contained in the motor, the displacement current density ∂D/∂t on the right side of Equation (1) can be neglected compared to the conduction current density J. In the derivation process, since the divergence of magnetic induction intensity B is always equal to zero, a vector magnetic potential A is introduced in the spinous magnetic field [17]:

(4) B=∇×A

The total conducted current density J is expressed [17] as

(5) $$J=J_{s}\times J_{e}$$

(6) $$J_{e}=\sigma E=-\sigma \frac{\partial A}{\partial t}$$

where $J_{s}$ is the source current density and $J_{e}$ is the eddy current density induced by the time variation of the magnetic field.

Finally, the 3-D eddy current field electromagnetic equation of the SPMLOA can be listed:

(7) $$v\nabla \times \nabla \times A=-\sigma \frac{\partial A}{\partial t}+J_{s}$$

where v is magnetic resistivity $\frac{1}{\mu }$. Since it is a 3-D field, the expression ∇×A in the Cartesian coordinate system is:

(8) $$\nabla \times A=\left|\begin{array}{ccc} i & j & k\\ \frac{\partial }{\partial x} & \frac{\partial }{\partial y} & \frac{\partial }{\partial z}\\ A_{x} & A_{y} & A_{z} \end{array}\right|=i\left(\frac{\partial A_{z}}{\partial y}-\frac{\partial A_{y}}{\partial z}\right)+j\left(\frac{\partial A_{x}}{\partial z}-\frac{\partial A_{z}}{\partial x}\right)+k\left(\frac{\partial A_{y}}{\partial x}-\frac{\partial A_{x}}{\partial y}\right)$$

In the cylindrical coordinate system:

(9) $$\nabla \times A=\left|\begin{array}{ccc} \alpha_{r}\frac{1}{r} & \alpha_{\theta } & \alpha_{z}\frac{1}{r}\\ \frac{\partial }{\partial r} & \frac{\partial }{\partial \theta } & \frac{\partial }{\partial z}\\ A_{r} & rA_{\theta } & A_{z} \end{array}\right|=\alpha_{r}\left(\frac{1}{r}\frac{\partial A_{z}}{\partial \theta }-\frac{\partial A_{\theta }}{\partial z}\right)+\alpha_{\theta }\left(\frac{\partial A_{r}}{\partial z}-\frac{\partial A_{z}}{\partial r}\right)+\alpha_{z}\left(\frac{1}{r}\frac{\partial \left(rA_{\theta }\right)}{\partial r}-\frac{1}{r}\frac{\partial A_{r}}{\partial \theta }\right)$$

This equation is obtained in a Cartesian coordinate system. For the calculation of the 3-D eddy current field of the SPMLOA, a solution area needs to be defined:

- For the stator slot area containing the armature winding, assuming $J=J_{s}$.
- The stator core is made of thin silicon steel sheets stacked together, and there is a certain air gap between the stacked sheets. Therefore, the eddy current loss in this area can be ignored, and the total current density and air gap area are the same J=0.
- Due to the presence of a solid permanent magnet and a supporting iron core, the moving sub region has a certain degree of conductivity, resulting in a current density in this area $J=-\sigma \frac{\partial A}{\partial t}$.
- The boundary conditions for each solution domain are A=0.

According to the variational principle, after a series of derivations, the corresponding functional is finally obtained:

(10) $$\left.\begin{array}{c} F\left(A\right)={∭}_{V}\left[\frac{v}{2}{\left(\nabla \times A\right)}^{2}+\frac{1}{2}\sigma \frac{\partial A}{\partial t}\cdot A-J_{s}\cdot A\right]dV=\min\\ A\left|\Gamma_{1}=0\right. \end{array}\right\}$$

### 3.2. Finite Element Calculation

**Figure 2:**
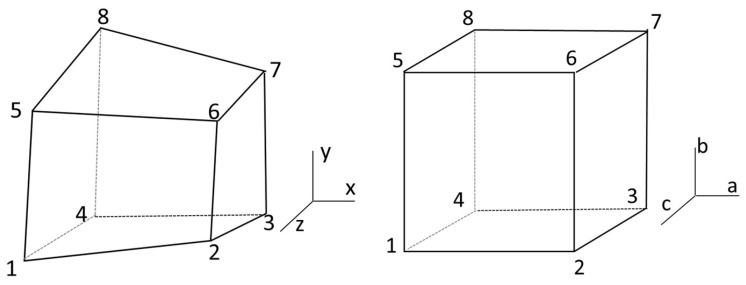
Hexahedral isoperimetric element.

The three most commonly used basic element partitioning methods for finite element calculations of 3-D eddy current fields are four-node tetrahedral elements, six-node pentahedral elements, and eight-node hexahedral elements. This article adopts the form of an eight-node hexahedral element, and the node numbers of the eight-node hexahedral element are shown in Figure 2. The relationship between the interpolation function and the basis function within the element is [18]:

(11) $$A\left(x,y,z\right)=\sum_{i=1}^{8}N_{i}A_{i}=\lambda_{1}+\lambda_{2}x+\lambda_{3}y+\lambda_{4}z+\lambda_{5}xy+\lambda_{6}yz+\lambda_{7}zx+\lambda_{8}xyz$$

where the basis function is

(12) $$N_{i}=\frac{1}{8}\left(1+a_{i}a\right)\left(1+b_{i}b\right)\left(1+c_{i}c\right)\begin{array}{cc} & \left(i=1,2,\cdots ,8\right) \end{array}$$

After deduction, the coefficient matrix of the unit can be obtained as:

(13) $$\begin{array}{l} \left[K_{lk}^{e}\right]={∭}_{V}\left\{v\left[\begin{array}{ccc} \frac{\partial N_{k}}{\partial y}\cdot \frac{\partial N_{l}}{\partial y}+\frac{\partial N_{k}}{\partial z}\cdot \frac{\partial N_{l}}{\partial z} & -\frac{\partial N_{k}}{\partial x}\cdot \frac{\partial N_{l}}{\partial y} & -\frac{\partial N_{k}}{\partial x}\cdot \frac{\partial N_{l}}{\partial z}\\ -\frac{\partial N_{k}}{\partial x}\cdot \frac{\partial N_{l}}{\partial y} & \frac{\partial N_{k}}{\partial x}\cdot \frac{\partial N_{l}}{\partial x}+\frac{\partial N_{k}}{\partial z}\cdot \frac{\partial N_{l}}{\partial z} & -\frac{\partial N_{k}}{\partial y}\cdot \frac{\partial N_{l}}{\partial z}\\ -\frac{\partial N_{k}}{\partial x}\cdot \frac{\partial N_{l}}{\partial z} & -\frac{\partial N_{k}}{\partial y}\cdot \frac{\partial N_{l}}{\partial z} & \frac{\partial N_{k}}{\partial x}\cdot \frac{\partial N_{l}}{\partial x}+\frac{\partial N_{k}}{\partial y}\cdot \frac{\partial N_{l}}{\partial y} \end{array}\right]+j\omega \sigma \left|\begin{array}{ccc} N_{l}N_{k} & & \\ & N_{l}N_{k} & \\ & & N_{l}N_{k} \end{array}\right|\right\}dV\begin{array}{cc} & \left(l,k=1,2,\cdots ,8\right) \end{array} \end{array}$$

The right vector of the unit is:

(14) $$\left[F_{l}^{e}\right]={∭}_{V}\left[\begin{array}{cc} \begin{array}{c} J_{sx}\\ J_{sy}\\ J_{sz} \end{array} & \begin{array}{c} N_{l}\\ N_{l}\\ N_{l} \end{array} \end{array}\right]dV\begin{array}{ccc} & & \left(l=1,2,\cdots ,8\right) \end{array}$$

By synthesizing the unit coefficient matrix as a whole and solving it, the magnetic potential values of each node can be obtained.

## 4. Electromagnetic Parameters

As stated earlier, the electromagnetic equations of the SPMLOA are solved using 3-D FEM to obtain the potential or magnetic potential of each node in the studied area. To obtain the final performance results of the SPMLOA, it is necessary to derive parameters such as magnetic field energy, electromagnetic force, inductance, and back EMF based on the obtained node potential or magnetic potential. This derivation process is generally referred to as post-processing of FEA.

### 4.1. Inductance

The SPMLOA studied in this paper only includes one phase winding. Its permanent magnet is located on the mover, providing the magnetomotive force of the main magnetic circuit. The magnetic field energy provided is [19]:

(15) $$W_{m}=\frac{1}{2}\int_{V}H\cdot BdV$$

According to Formula (4), the magnitude of the magnetic field energy can ultimately be calculated. According to the relationship between magnetic field energy and inductance:

(16) $$W_{m}=\frac{1}{2}LI^{2}$$

The inductance is given:

(17) $$L=\frac{2W_{m}}{I^{2}}$$

### 4.2. Magnetic Flux

Flux linkage refers to the magnetic flux of the links in the armature winding, which is equal to the product of the number of turns in the coil and the average magnetic flux passing through each turn of the coil. In 3-D finite element calculations, the magnetic flux passed through the winding coil is [19]:

(18) $$d\Psi =\frac{NdS}{S}\Phi =\frac{NdS}{S}\oint_{l}Adl$$

where N is the number of turns of the winding, S is the total cross-sectional area of the winding, and l is the length of a single winding coil. To calculate the total magnetic flux passing through the winding, the above equation is integrated over the total area:

(19) $$\Psi =\int_{S}d\Psi =\frac{N}{S}\int_{S}\left(\oint_{l}A\cdot dl\right)dS=\frac{N}{S}\int_{V}A\cdot l_{0}dV$$

where $l_{0}$ is unit vector along the direction of differential length vector dl, and V is the volume of the winding area.

### 4.3. Induced Electromotive Force

According to Faraday’s law of electromagnetic induction, the cross chain magnetic flux of the armature coil winding generates induced voltage, also known as induced EMF or back EMF, at both ends of the armature coil winding. The formula is [19]:

(20) $$E=-\frac{d\Psi }{dt}=-\frac{d\Psi }{dx}\frac{dx}{dt}=-v_{s}\frac{d\Psi }{dx}$$

where $v_{s}$ is the speed of the mover, and x is the displacement of the mover.

### 4.4. Electromagnetic Force

The calculation of electromagnetic thrust based on the principle of virtual work is the differentiation of magnetic field energy with respect to the displacement of the moving body. The calculation formula for magnetic field energy has been given above, so the electromagnetic thrust received by the rotor in the z-direction is [19]:

(21) $$F_{Z}=-\frac{\partial W_{m}}{\partial z}={∭}_{V}\left(B\frac{dH}{dz}\right)dV+{∭}_{V}\left(B\cdot dH\right)\frac{\partial }{\partial z}dV$$

It is difficult to derive an analytical formula for Equation (21), so numerical calculation methods can only be used to determine the electromagnetic thrust acting on the object. Using the FEM, the discretized electromagnetic thrust can be expressed as:

(22) $$F=\sum_{j=1}^{m}F_{j}=\sum_{j=1}^{m}\left(\sum_{j=1}^{n}H_{ei}^{T}M_{ei}H_{ei}\right)$$

where $F_{j}$ is the electromagnetic thrust of the node j, m is the total number of the nodes, n is the number of units connected to the node j, $H_{e}$ is the magnetic field strength matrix of the unit nodes, and $M_{e}$ is the symmetry coefficient matrix, which can be expressed as:

(23) $$M_{e}=-3\mu_{e}^{T}\int N_{e}^{T}N_{e}\frac{\partial N_{e}^{T}}{\partial z}d\Omega +\frac{\mu_{e}^{T}}{J_{o}}\frac{\partial J_{o}}{\partial z}\int N_{e}^{T}N_{e}N_{e}^{T}d\Omega$$

where $\mu_{e}$ is the magnetic permeability matrix, $N_{e}$ is the unit shape function matrix, and $J_{o}$ is the Jacobian matrix.

## 5. Comparison of Simulation Results

**Figure 3:**
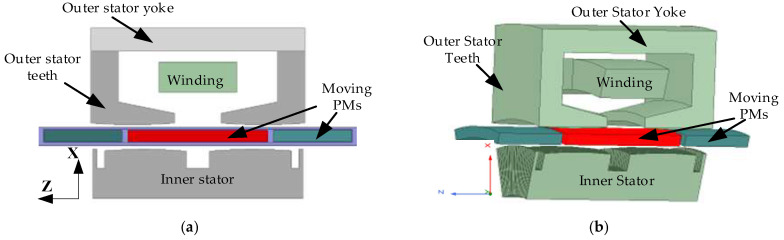
Finite element models of the novel SPMLOA: (**a**) 2-D; (**b**) 3-D.

Due to the fact that the laminations of the SPMLOA are usually stacked along the circumferential direction to reduce magnetic resistance, 3-D finite element modeling is used for accuracy in calculations. In this section, 2-D and 3-D finite element simulation software Ansoft Maxwell (https://www.ansys.com/products/electronics/ansys-maxwell, accessed on 28 November 2024) is used to model and analyze the SPMLOA. Firstly, the 2-D and 3-D models of the SPMLOA are established, as shown in Figure 3. The size specifications and technical parameters of this novel SPMLOA are listed in Table 1. On this basis, the static performance of the SPMLOA is specifically analyzed, including magnetic field distribution, no-load permanent magnet flux, no-load back EMF, winding inductance, detent force, electromagnetic force, etc.

### 5.1. Magnetic Field Distribution

**Figure 4:**
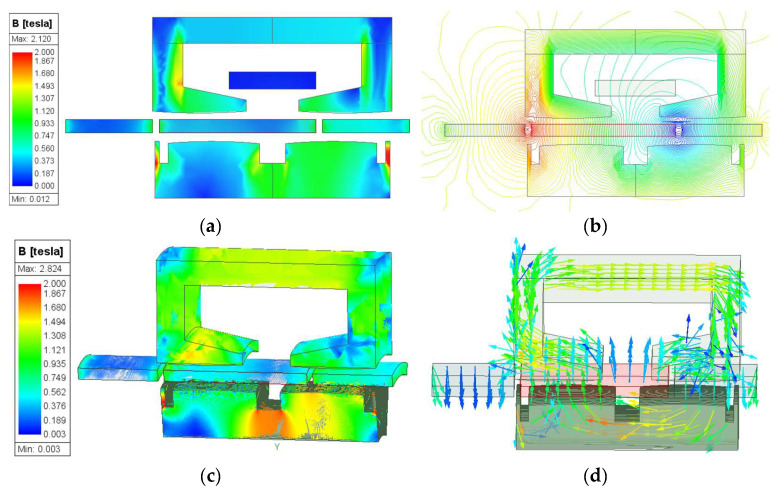
Magnetic field distribution of the SPMLOA at maximum displacement: (**a**) 2-D magnetic field density; (**b**) 2-D magnetic circuit; (**c**) 3-D magnetic field density; (**d**) 3-D magnetic field density.

Figure 4 shows the open circuit magnetic field distribution predicted by FEM when the mover is at maximum displacement with a 2-D model and 3-D model. The magnetic field saturation region of the SPMLOA is mainly concentrated near the inner stator auxiliary slot. Due to the fact that the 3-D model is built based on an actual prototype structure, its magnetic field saturation is significantly greater than that of the 2-D ideal model, as shown in Figure 4a,c. The magnetic density vector in the 3-D model is used to display the distribution of the main magnetic circuit. According to the magnetic field distribution map, it can be seen that there is a significant leakage of magnetic flux in the permanent magnet of the SPMLOA during operation, which is also the main problem with this type of SPMLOA.

### 5.2. No-Load Permanent Magnet Flux

**Figure 5:**
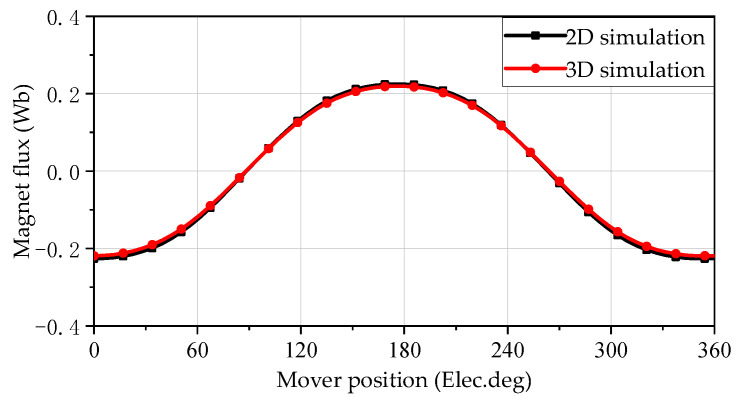
Flux linkage waveform of open-circuit.

The mover of the SPMLOA operates at a sinusoidal speed with a frequency of 20 Hz, and the other parameters are the same. Two dimensional and 3-D models of the SPMLOA are simulated under no-load conditions to obtain a comparative model of the magnetic flux, as shown in Figure 5. It can be seen that the waveform of the 2-D model simulation results is smoother, mainly because the 2-D simulation model has more subdivision units and more accurate data. At the same time, the amplitude of the magnetic flux generated by the 2-D simulation is significantly larger than the 3-D simulation results, because of the special stacking method of the SPMLOA; there are corresponding gaps between each silicon steel sheet during the assembly of the inner stator silicon steel sheet, and the 2-D model cannot effectively reproduce it, so it differs greatly from the actual situation. The 3-D simulation results are closer to the actual situation.

### 5.3. No-Load Back Electromotive Force

**Figure 6:**
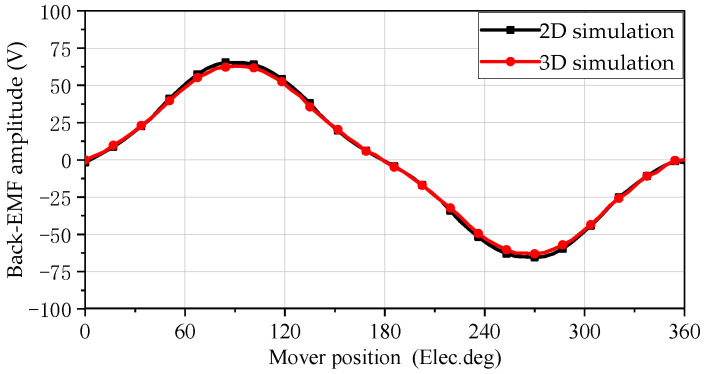
Back EMF waveform of open-circuit.

The no-load induced EMF is one of the main parameters for measuring the performance of a motor, and the quality of its waveform has a significant impact on both the motor output and no-load. The no-load induced EMF of the SPMLOA at sinusoidal speed is shown in Figure 6. From the waveform, it can be seen that the waveform shape of the induced EMF obtained by the two simulation methods is consistent. Similar to the magnetic flux simulation results, the back EMF amplitude obtained from the 2-D simulation is 64.4 V, which is slightly larger than the 3-D simulation results.

### 5.4. Detent Force

**Figure 7:**
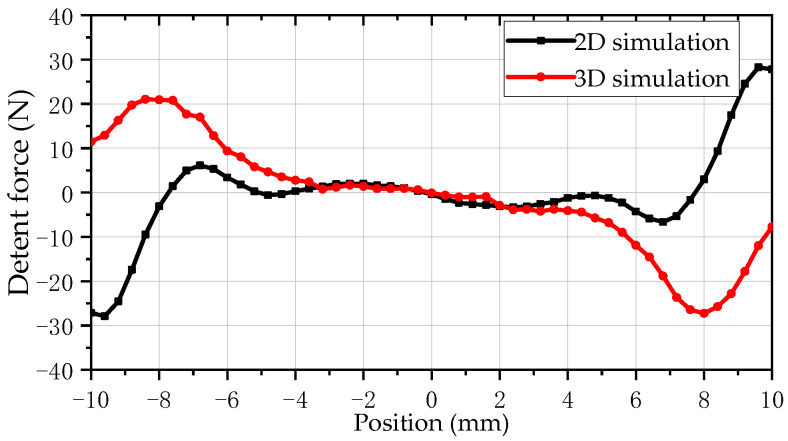
Comparison of detent forces.

Figure 7 compares and analyzes the detent force waveforms obtained by two models. The mover of the SPMLOA is set to operate at a constant speed of 1 m/s, with an armature current of 0A and a stroke of 20 mm. As shown in the figure, the detent force amplitude of the 2-D model simulation is 10 N at the effective stroke, while the 3-D finite element simulation result is 21 N. Compared to 2-D simulation results, the detent force waveform obtained from 3-D simulation exhibits greater fluctuations. This is mainly due to the ideal 2-D model, which cannot accurately obtain the true results of the SPMLOA due to the process errors in the assembly of the stator laminations. Therefore, the detent force waveform obtained from 3-D model simulation is closer to that of the actual prototype.

Through the calculation of the electromagnetic characteristics of the PMLOA motor mentioned above, it was found that the 2-D FEA has a significantly faster calculation speed than the 3-D FEA. The desktop computing performance used for simulation was as follows: CPU frequency: 4.66 GHz, and memory capacity: 32 GB. The partitioning and calculation times of the two simulation models are shown in Table 2. From the table, although the number of elements in the 2-D model is relatively small, its result error rate is small, the simulation time is fast, and it is suitable for large-scale optimization calculations. However, there are significant fluctuations in the calculation of detent force using 2-D models, so it is necessary to compare and verify the calculation results using 3-D models.

## 6. Experimental Verification

**Figure 8:**
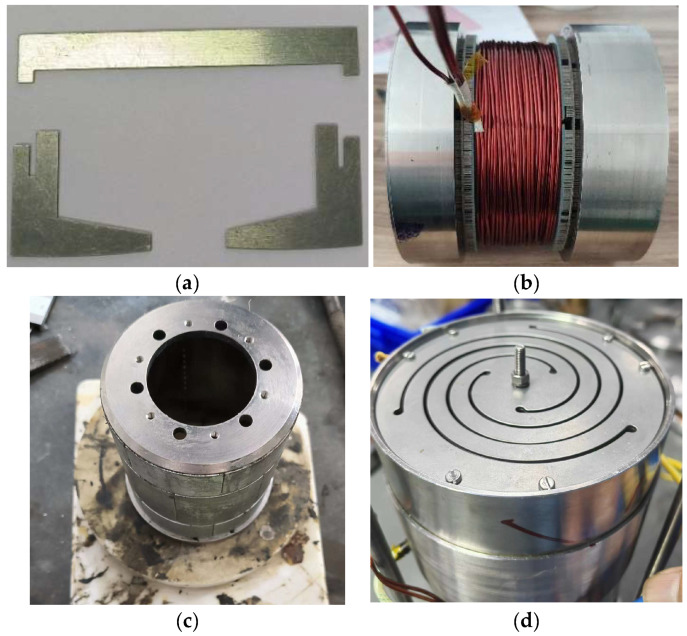
Assembly process of the prototype: (**a**) outer stator tooth silicon steel sheet; (**b**) s winding coil; (**c**) mover with PMs; (**d**) overall structure of the LOA.

The SPMLOA prototype proposed in this paper was machined and assembled to verify the FEA results, as shown in Figure 8. It can be seen that the outer stator teeth and yoke of the prototype are separately processed from silicon steel sheets. During assembly, the outer stator teeth are first assembled by circumferential stacking, then the insulation slot is simulated to wind the winding coil. This method effectively reduces the difficulty of placing the concentrated winding, as shown in Figure 8b. In addition, the mover permanent magnet is fixed at both ends of the SPMLOA housing through two plate springs to support the movement of the mover, as shown in Figure 8d. This installation method does not require end caps and bearings, which not only effectively reduces the quality of the SPMLOA, but also increases its heat dissipation capacity.

**Figure 9:**
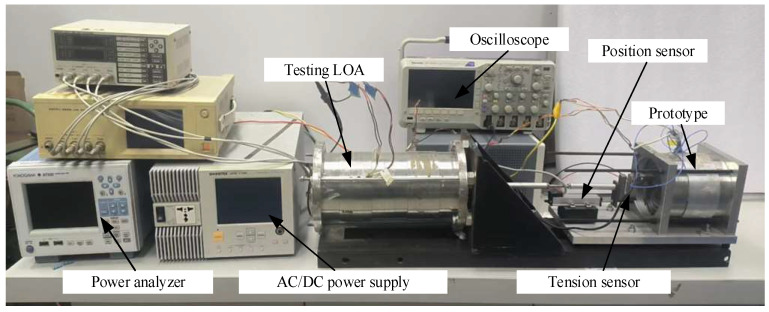
Test platform of the proposed novel SPMOA.

Figure 9 shows the SPMLOA prototype testing platform, which mainly includes a YOKOGAWA (Tokyo, Japan) WT500 power analyzer, programmable AC/DC power supply, oscilloscope, the prototype, a testing LOA and tension sensor. A programmable AC/DC power supply is mainly used to regulate the voltage value and frequency of the output voltage. Two LOAs are connected to tension sensors and position sensors through screw rods. The tension sensor causes a change in the oscilloscope test voltage through its own deformation, thereby displaying the corresponding tension value. By adjusting the operating mode of the testing LOA, the prototype can be driven to achieve corresponding electromagnetic characteristic testing.

**Figure 10:**
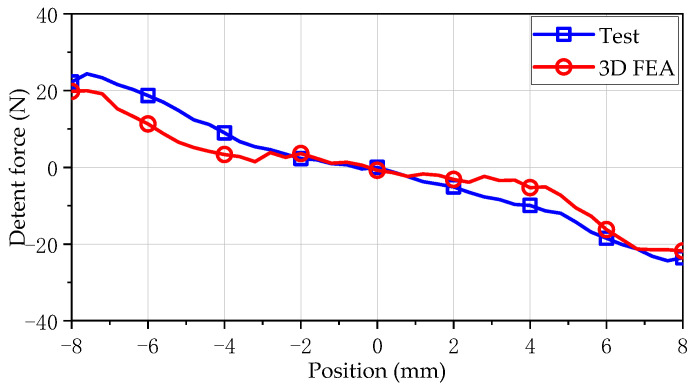
Comparison results of testing and 3-D simulation of detent force waveform under no-load conditions.

Figure 10 compares the test results of the detent force at a speed of 0.5 m/s with the 3-D simulation results. The 3-D FEA results align well with the experimental results, with values of 20 N and 24 N, respectively. In the process of 3-D simulation, the accuracy of mesh division affects the calculation speed and result accuracy, so it is necessary to set the number of mesh division reasonably. Although the detent force of the actual prototype has not yet achieved an optimal suppression effect, it marks a notable advancement when compared to the original model.

**Figure 11:**
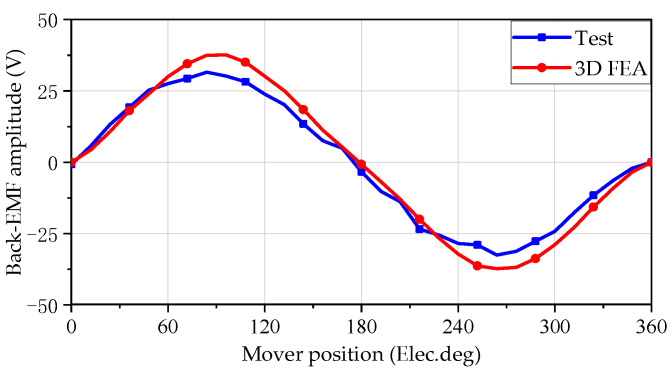
Comparison results of testing and 3-D simulation of back electromotive force waveform under no-load conditions.

The back electromotive force (EMF) is the most easily obtainable and effective electromagnetic characteristic indicator that reflects whether the prototype performance meets the simulation design requirements. In order to obtain the results of the back EMF test, the output AC voltage of the testing LOA from the programmable AC/DC power supply is 10 V, 20 Hz, so that the testing LOA can drive the prototype to achieve a motion with a stroke of 8 mm and a frequency of 20 Hz. Figure 11 shows the comparison waveform of the 3-D simulation and test back electromotive force with a frequency of 20 Hz. Evidently, the tested back EMF waveform exhibits a sinusoidal pattern, aligning seamlessly with the 3-D calculations. Specifically, the amplitudes of the back EMF, as observed in both testing and 3-D calculations, are 31 V and 35 V, respectively. The primary factor contributing to the back EMF of the prototype being relatively low lies in the separate assembly of the outer teeth and yoke. This assembly method leads to an augmentation in the magnetic resistance within the magnetic circuit. Furthermore, inaccuracies in the installation of the inner and outer stators and mover during the assembly process can also detract from its overall performance.

Usually, linear oscillating motors operate in a mechanical resonance frequency, which is associated with the overall spring stiffness K of the mover and the mass M of the mover system. Based on the analysis of the dynamic model for LOA, the overall spring stiffness of the LOA encompasses both the stiffness of the spring and the equivalent gas spring stiffness [20,21]. Consequently, the formula for calculating the mechanical resonance frequency of the motor is as follows:

(24) $$\omega_{m}=\sqrt{\frac{K}{M}}=\sqrt{\frac{k_{m}+k_{g}}{M}}$$

where $k_{m}$ is the spring stiffness of the mover and $k_{g}$ is the gas equivalent spring stiffness.

Generally, when the LOA operates at the mechanical resonance point, the efficiency of the SPMLOA is highest, and its effective stroke is maximized at the same voltage. Therefore, before conducting load experiments on the LOA, it is necessary to obtain the resonance frequency of the LOA during operation by sweeping the frequency.

**Figure 12:**
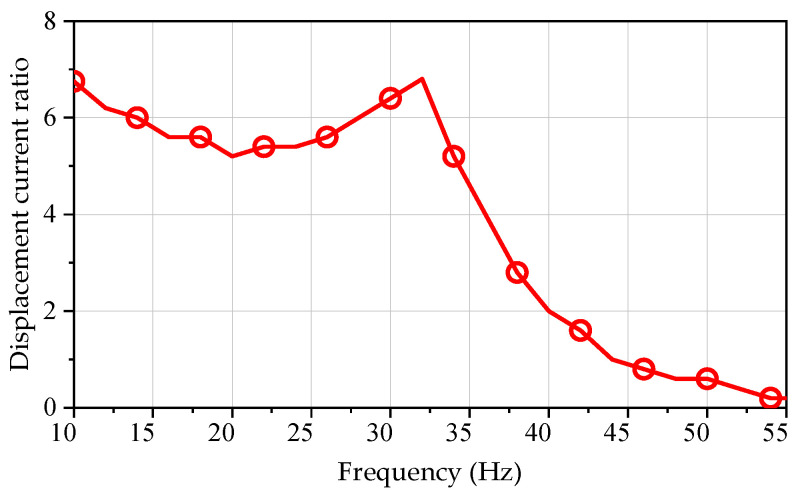
Relationship between displacement current ratio and frequency under no-load state.

Firstly, a programmable AC/DC power supply is used to conduct no-load resonance frequency testing on the prototype, with a sweep frequency range of 10~55 Hz and a step size of 2 Hz. The displacement and current values inside the motor are tested using position sensors and current clamps. Figure 12 shows the relationship between displacement current ratio and mechanical frequency of the SPMLOA prototype under no-load condition. As shown in the figure, the fluctuation of the sweeping curve of the prototype first decreases, then increases, and then decreases again. When the frequency is 32 Hz, the displacement current ratio of the prototype reaches its maximum value. According to the waveform it can be seen that, when the operating frequency is lower than the resonant frequency, the magnitude of the displacement current ratio remains high, and its minimum value only decreases by 24% compared to the resonant frequency. When the operating frequency is greater than the resonant frequency, the displacement current ratio sharply decreases. Therefore, the operating frequency of the linear vibration motor should be controlled within a certain range below the resonant frequency. At the same time, due to the fact that the elastic force of the plate spring used in the prototype is lower than the design value, and the influence of the gas spring on the resonance frequency is eliminated in the unloaded state, the unloaded frequency of the prototype is lower than the design requirement of 40 Hz.

**Figure 13:**
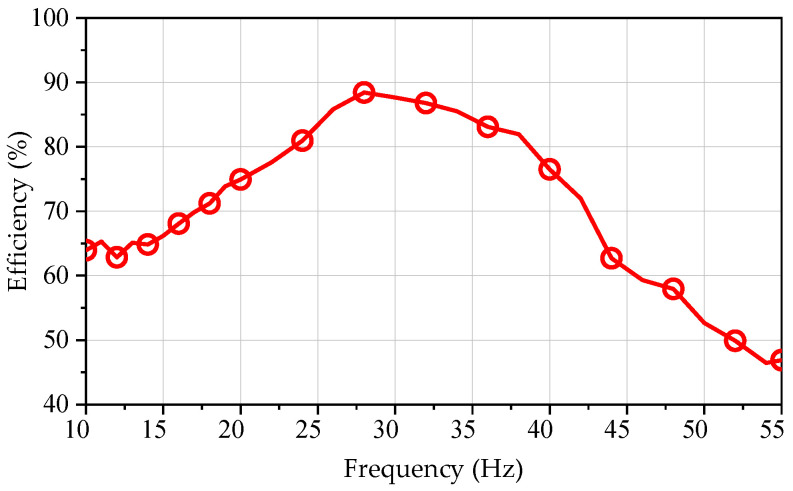
Efficiency waveform of the prototype at different frequencies.

The prototype drags the load LOA for load testing, with the prototype in electric mode and the load LOA in power generation mode [22]. The input power of the LOA is maintained at 200 W and operated at a frequency of 10~55 Hz. Figure 13 illustrates the relationship between the efficiency of the SPMLOA prototype and frequency. As the frequency of the mover increases, the efficiency of the prototype initially rises and then falls. The prototype achieves a maximum efficiency of 88.4% at a frequency of 28 Hz. When the frequency exceeds 40 Hz, the efficiency of the prototype sharply decreases, primarily due to increased eddy current losses inside the motor at higher frequencies. However, the power tested in this study is relatively low, leading to a significant decrease in efficiency. Compared to the resonance frequency measured under no-load conditions, since the two LOAs are in a paired state at this time, they can be regarded as a whole, and the resonance frequency of the paired system is different from that of a single LOA.

## 7. Conclusions

This article proposes a new type of single-phase permanent magnet linear oscillation actuator, which uses a C-shaped iron core for the outer stator and auxiliary slots and arc-shaped teeth for the inner stator to achieve smaller positioning forces. The article introduced the basic structure of the SPMLOA. According to the FEM, the function of the SPMLOA is listed, and the listed function is calculated using an eight-node hexahedral element form. In addition, this article compared and analyzed the electromagnetic characteristics obtained from the simulation of 2-D finite element models and 3-D finite element models. The results showed that the 2-D simulation time is only 2% of the 3-D simulation time, and the error between the two is less than 5%. However, due to the special structure of this SPMLOA, there is a 12% error between the simulation results and the actual test results. Finally, the test results indicate that the no-load resonant frequency of the SPMLOA motor is 32 Hz. When the frequency is 28 Hz, the maximum efficiency of the SPMLOA motor is 88.4%.

## Figures and Tables

**Table 1:** The dimension parameters of the SPMLOA.

Parameter	Value	Unit	Parameter	Value	Unit
Output power	200	W	Outer stator height	25.5	mm
Rated voltage	60	V	Back iron height	6	mm
Rated frequency	40	Hz	Slot width	12	mm
Rated stroke	±8	mm	Air gap	1.5	mm
Thrust	200	N	Inner-stator height	13	mm
Efficiency	85%		PM height	3	mm
Stator length	55	mm	Middle PM width	36	mm
Outer-stator inner diameter	39.5	mm	Right and left PM width	20	mm

**Table 2:** Comparison of efficiency between two simulation methods.

Parameter	2-D	3-D
Total number of elements	22,448	333,526
Simulation time	5 min	5 h 34 min
Error rate	<5%	

## Data Availability

The original contributions presented in this study are included in the article. Further inquiries can be directed to the corresponding author.
